# Relationship between glycemic intraday variations evaluated in continuous glucose monitoring and HbA1c variability in type 2 diabetes: pilot study

**DOI:** 10.1186/s13098-021-00663-2

**Published:** 2021-04-15

**Authors:** Akemi Tokutsu, Yosuke Okada, Keiichi Torimoto, Yoshiya Tanaka

**Affiliations:** First Department of Internal Medicine, School of Medicine, University of Occupational and Environmental Health, 1-1 Iseigaoka, Yahatanishi-ku, Kitakyushu, 807-8555 Japan

**Keywords:** HbA1c variability, Flash glucose monitoring (FGM), Hypoglycemia

## Abstract

**Background:**

HbA1c variability is independent of mean HbA1c, and associated with mortality due to vascular complications. However, the significance of HbA1c variability is unknown at present. In this study, we used flash glucose monitoring (FGM) and evaluated glycemic intraday variations, and then examined the association with HbA1c variability.

**Methods:**

We conducted a retrospective pilot study of 26 patients treated at the Outpatient department for type 2 diabetes mellitus (T2DM), and evaluated the following items associated with blood glucose levels and their changes/variations using FGM. The primary endpoint was factor(s) associated with standard deviation (SD) HbA1c over a 6-month period. To adjust for the effect of varying numbers of HbA1c measurements, we used the adjusted SD of HbA1c.

**Results:**

There were significant correlations between mean HbA1c and each of glucose management indicator, maximum, percent time at glucose > 180 mg/day, mean of daily difference of blood glucose, and high blood glucose index. Adjusted SD HbA1c correlated significantly with percent time at glucose < 70 mg/dL and low blood glucose index. We estimated the regression coefficient of adjusted SD HbA1c using multivariate linear regression analysis, and noted that the presence of hypoglycemia affected Adjusted SD HbA1c (β = 0.130, SE = 0.044, P = 0.008). Hypoglycemia was noted in 17 patients, and adjusted SD HbA1c was significantly higher (p = 0.001) in the hypoglycemic group (0.22 ± 0.12%), compared with the non-hypoglycemic group (0.08 ± 0.05%). The cut-off value of adjusted SD HbA1c was 0.109% in the hypoglycemic group.

**Conclusions:**

The results showed that HbA1c variability is associated with hypoglycemia. For patients with high HbA1c variability, we recommend evaluation for the presence of hypoglycemia and reconsideration of their treatment regimen including their glucose-lowering medications.

*Trial registration*

The study protocol and opt-out method of informed consent were approved by the ethics committees of the University of Occupational and Environmental Health (Trial registration: H27-186, Registered 25 Dec 2015)

**Supplementary Information:**

The online version contains supplementary material available at 10.1186/s13098-021-00663-2.

## Background

One of the objectives of treatment of diabetes is prevention of future diabetes-related complications through glycemic control. HbA1c is the gold standard index used for monitoring the effectiveness of treatment of diabetes. HbA1c reflects blood glucose level over the previous 3 months, and it is not affected by short periods of blood glucose variations, such as changes following meals and exercise [1, 2]. In this regard, Continuous Glucose Monitoring (CGM) and Flash Glucose Monitoring (FGM) have been recommended for managing more specific glucose levels, and the time in range (TIR) and blood glucose levels of 180 or above and under 70 have become international standards for monitoring the response to diabetes treatment [3]. While CGM is a useful supplementary device, it is difficult to use in all patients during routine clinical practice.

Clinical evidence indicates that keeping HbA1c < 7% is beneficial as it prevents the development of microangiopathy. The Diabetes Control and Complications Trial (DCCT) group has reported that the use of HbA1c as an index of glycemic control slows down the progression of diabetic retinopathy, nephropathy and neuropathy [4]. Furthermore, the effects of intensive glucose-lowering therapy on blood pressure and albumin excretion were still evident at 7 to 8 years after the end of the DCCT study; in the intensive therapy group, the odds ratio was 59% for the prevention of nephropathy and 84% for the prevention of progression to overt albuminuria. In this regard, a previous study of DCCT with 22-year follow-up reported 50% risk reduction in GFR (< 60 mL/min/1.73 m^2^) in the intensive therapy group, coupled with significant reduction in the GFR decline rate [5, 6]. Furthermore, the United Kingdom Prospective Diabetes Study (UKPDS) showed that for each 1% drop in HbA1c, the risk of microvascular complications reduces by 37% [7]. These trials verified the importance of elevated HbA1c as an indicator of chronic hyperglycemia. Another detailed blood glucose profile study using CGM concluded that HbA1c reflects the mean blood glucose level [8].

The ADVANCE trial [9] examined the effect of glycemic control, using HbA1c as the index parameter, and reported that intensive glucose-lowering therapy was not associated with a significant reduction in macrovascular disorders. Furthermore, the ACCORD trial concluded that intensive therapy was associated with significantly higher all-cause mortality and cardiovascular mortality hazard ratios [10]. These studies suggest that serious hypoglycemia linked to intensive therapy may underlie the increased risk of mortality. In this regard, recent studies have reported that large swings in blood glucose levels are associated with worsening of arteriosclerosis [11] and increased risk of mortality due to cardiovascular events [12]. Apart from blood glucose levels, the correlation between HbA1c variability, in addition to mean HbA1c, and mortality due to diabetic vasculopathies has also been analyzed [13–15]. However, to date, the true significance of HbA1c variability remains unknown.

We used the FGM system to examine the relationship between HbA1c variability and detailed glycemic profiles. The primary endpoint was the identification of background parameters and CGM indices that are associated with adjusted SD HbA1c. The latter term represents the variability of HbA1c over a 6-month period. The secondary endpoint was the identification of background parameters and CGM indices that are associated with the mean HbA1c over the 6-month period**.**

## Methods

### Subjects

We conducted a retrospective study from September 2018 to January 2019 at the Outpatient Clinic of the University of Occupational Medicine Hospital and University of Occupational Medicine Wakamatsu Hospital. The subjects were 26 patients with type 2 diabetic mellitus (T2DM), who had been on the FGMS® System (FreeStyle Libre Pro System, Abbott Diabetes Care, Inc.) for at least eight days for evaluation of hemodynamics. The following inclusion criteria were applied in this study: (1) age between 30 and 80 years at the time of obtaining consent; (2) T2DM treated and followed-up at the Outpatients Clinic; (3) no changes (addition, switching, or discontinuation) to the glucose-lowering agents or their doses within the 4-week period before the commencement of the FGMS; (4) no changes to the T2DM treatment up to 6 months after the start of FGM. The following exclusion criteria were also applied. (1) Type 1 or secondary diabetes mellitus; (2) severe infection, before- or after surgery, or serious trauma; (3) renal dialysis; (4) severe hepatic dysfunction (AST ≥ 100 IU/L or ALT ≥ 100); (5) moderate or severe heart failure (NYHA/New York Heart Association Classification III or higher stage); (6) pregnant, lactating, or potentially pregnant patients; (7) treated with steroids or other drugs that affect blood glucose levels; and 8) history of macrovascular diseases. The following definitions were used for diabetic microangiopathies. Diabetic neuropathy was diagnosed by the presence of two or more clinical symptoms (bilateral spontaneous pain, hypoesthesia or paresthesia of the legs), absence of ankle tendon reflexes and decreased vibration sensations using a C64 tuning fork. Diabetic retinopathy was defined as mild or severe retinopathy based on the results of funduscopic examination by ophthalmologists. Diabetic nephropathy was defined as albumin-to-creatinine ratio ≥ 30 mg/g creatinine.

The study protocol and opt-out method of informed consent were approved by the ethics committees of the University of Occupational and Environmental Health (Trial registration: H27-186, Registered 25 Dec 2015).

### Flash glucose monitoring system

The following parameters were measured using the data recorded by the FGM: average glucose level (AG), standard deviation (SD), coefficient of variation (CV), percent time at glucose level of 70–180 mg/dL (TIR: time in range), percent time at > 180 mg/dL (TAR: time above range), percent time < 70 mg/dL (TBR: time below range), maximum, minimum, glucose management indicator (GMI) [16], mean daily difference of blood glucose (MODD), low blood glucose index (LBGI) and high blood glucose index (HBGI) [17]. Hypoglycemia was defined as a glucose value less than 70 mg/dL as recorded by FGM. FGM was applied once at the start of the study and used for up to 14 days. The FGM data were recorded to avoid bias due to the insertion and removal of the FGM, or lack of stability of the unit. Since the MARD, which represents the accuracy of the sensor in FGM, is higher on the first day [18], and since most patients followed weekly routines in their daily living, we used data obtained from the second to seven days, and excluded those of the first day. We recorded the daily average value, and listed the average value for seven days. The mean percentage of time during which the FGM was active for all 26 cases was 97.4%, and the analysis included no missing values over the 7-day period.

### Laboratory tests

HbA1c (%) was measured by HPLC using Tosoh HLC-723 G8 (Tosoh Co., Kyoto, Japan) and recorded as a NGSP (National Glycohemoglobin Standardization Program) value. eGFR (estimated glomerular filtration rate) was calculated as 194 × serum creatinine concentration (mg/dL) − 1.094 × age − 0.287 for men, and 194 × serum creatinine concentration (mg/dL)  − 1.094 × age − 0.287 × 0.739 for women [19]. The SD HbA1c represents the SD of 3–7 HbA1c readings over a 6-month period from the time of starting the use of FGM. The SD HbA1c represents the SD of 3–7 HbA1c readings over a 6-month period from the time of starting FGM use. The median number of HbA1c measurements was 4.5. To adjust for the effect of different numbers of HbA1c measurements, we used in this study the adjusted SD of HbA1c, representing the SD of HbA1c divided by [*n*/(*n*–1)]^0.5^, where *n* is the number of HbA1c measurements [20]. On the other hand, the mean HbA1c reported in this study is the mean of the same measurements.

### Statistical analysis

Continuous values are shown as mean values and categorical variables are expressed as count and percentage values. The Shapiro–Wilk test was used to test for normality; for statistical significance of the mean values of two groups, the Student's t-test was used if equal variance was confirmed by the F test, whereas Welch's t-test was used if it followed normal distribution. The Mann–Whitney U test was used if it did not follow a normal distribution, and Spearman's correlation analysis was used for testing the relationship between two variables. Univariate and multivariate linear regression analyses were used to estimate the regression coefficients for adjusted SD HbA1c. Multivariate analysis was performed with adjusted SD HbA1c as the dependent variable, and age, BMI, and presence of hypoglycemia as independent variables. Dummy variables were created for gender and presence of hypoglycemia, and then used in multiple regression analysis. The cutoff value of adjusted SD HbA1c was examined based on the ROC curve. The calculated sample size in the ROC analysis was 30 patients in total, assuming an area under the curve (AUC) of 0.80, power 0.80, with significance level of 5%, and a non-hypoglycemic group versus the hypoglycemic group of 2:1. A *p* value < 0.05 was considered significant. All analyses were performed using SPSS Statistical Software 25.0 (SPSS Inc., Chicago, IL).

## Results

### Clinical characteristics of study participants

The clinical characteristics are shown in Table 1. The study participants were 26 patients (14 males, 12 females) aged 68.5 ± 7.8 years (range 51–79 years), with BMI of 24.3 ± 4.1 kg/m^2^, baseline HbA1c 6.7 ± 0.6% (5.7–7.7%), adjusted SD HbA1c 0.17 ± 0.12% (0.00–0.47%), mean HbA1c 6.8 ± 0.6% (5.7–7.8%), and history of diabetes of 12.6 ± 10.0 years (1.3–30 years). The prevalence of microangiopathy was 19% for neuropathy, 8% for retinopathy, and 23% for nephropathy. The most commonly used antidiabetic drug was DPP-4 inhibitor at 65%, followed by biguanide at 46%. None of the patients developed serious hypoglycemic events throughout the study period.

**Table 1:** Baseline characteristics and CGM parameters of the 26 patients

	Mean ± SD
Sex (male/female)	14/12
Age (year)	68.5 ± 7.8
Height (cm)	159.9 ± 8.2
Weight (kg)	62.5 ± 13.9
BMI (kg/m^2^)	24.3 ± 4.1
SBP (mmHg)	136.4 ± 16.8
DBP (mmHg)	73.7 ± 9.6
Duration of diabetes (years)	12.6 ± 10.0
Baseline HbA1c (%)	6.7 ± 0.6
Creatinine (mg/dL)	0.85 ± 0.23
eGFR (mL/min/1.73m^2^)	63.9 ± 14.2
Neuropathy [n (%)]	5 (19)
Retinopathy [n (%)]	2 (8)
Nephropathy [n (%)]	6 (23)
Diabetes treatment	
Diet only [n (%)]	1 (4)
SU [n (%)]	0
Glinide [n (%)]	2 (8)
DPP-4 inhibitor [n (%)]	17 (65)
Biguanide [n (%)]	12 (46)
Thiazolidine [n (%)]	5 (19)
SGLT-2 inhibitor [n (%)]	7 (27)
α-glucose inhibitor [n (%)]	5 (19)
GLP-1 receptor [n (%)]	3 (12)
Insulin [n (%)]	2 (8)
CGM parameters	
Average glucose (mg/dL)	129.2 ± 21.1
MODD (mg/dL)	27.5 ± 9.5
SD (mg/dL)	33.1 ± 8.7
CV (%)	25.6 ± 4.7
Maximum (mg/dL)	214.0 ± 36.8
Minimum (mg/dL)	81.3 ± 15.4
TBR (%)	1.5 ± 2.5
TIR (%)	87.1 ± 11.8
TAR (%)	11.4 ± 11.8
LBGI	1.6 ± 1.1
HBGI	4.2 ± 2.3

BMI: Body mass index; SBP: systolic blood pressure; DBP: diastolic blood pressure; eGFR: estimated glomerular filtration rate; SU: sulfonylureas; DPP-4 inhibitor: dipeptidyl peptidase-4 inhibitor; SGLT-2 inhibitor: sodium-glucose transporter-2 inhibitor; GLP-1 receptor: Glucagon-like peptide-1 receptor; CGM: continuous glucose monitoring; MODD: mean of daily difference of blood glucose; SD: standard deviation; CV: coefficient of variation; TBR: time below range; TIR: time in range; TAR: time above range; LBGI: low blood glucose index; HBGI: high blood glucose index

### Factors associated with adjusted SD HbA1c

**Fig. 1:**
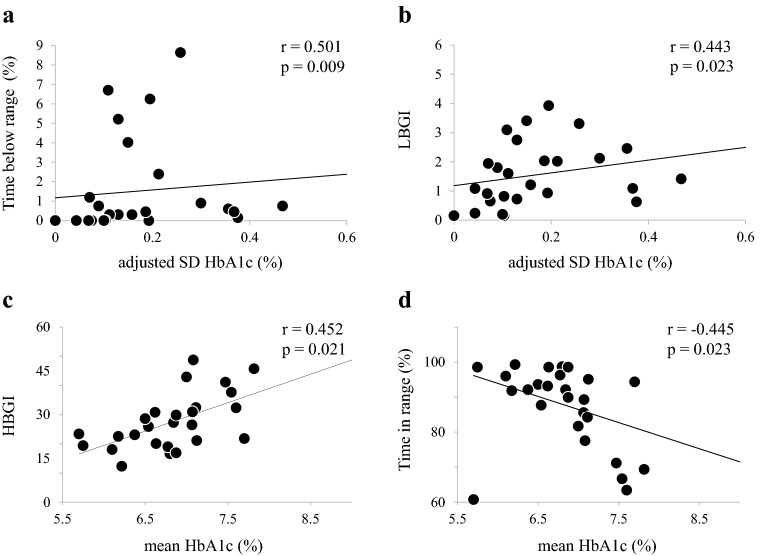
Correlations between adjusted SD HbA1c or mean HbA1c and CGM metrics. Data were obtained from Spearman’s correlation analysis. SD: standard deviation; TBR:Time below range; LBGI: low blood glucose index; HBGI: high blood glucose index; TIR: Time in range

Table 2 shows the correlation between adjusted SD HbA1c and each of the listed factors. There was significant correlation between adjusted SD HbA1c and TBR (r = 0.501, P = 0.009; Fig. 1a), and between adjusted SD HbA1c and LBGI (r = 0.443, P = 0.023; Fig. 1b). There was no significant difference in adjusted SD HbA1c between with and without each drug (Additional file 1: Table S1).

**Table 2:** Correlation with adjusted SD HbA1c and mean HbA1c

	Adjusted SD HbA1c		Mean HbA1c	
	ρ	P value	ρ	P value
Average glucose (mg/dL)	− 0.242	0.233	0.624	0.001*
SD (mg/dL)	0.023	0.912	0.463	0.017*
CV (%)	0.277	0.171	0.266	0.190
GMI (%)	− 0.242	0.233	0.624	0.001*
Maximum (mg/dL)	− 0.202	0.323	0.467	0.016*
Minimum (mg/dL)	− 0.369	0.063	0.319	0.113
TBR (%)	0.501	0.009*	− 0.184	0.369
TIR (%)	0.139	0.499	− 0.445	0.023*
TAR (%)	− 0.239	0.239	0.525	0.006*
MODD (mg/dL)	− 0.05	0.809	0.570	0.002*
LBGI	0.443	0.023*	− 0.165	0.421
HBGI	− 0.145	0.480	0.452	0.021*

The FGM data used seven consecutive days, and the values in the table represent the average of 7 days

Data are results of Spearman rank correlation. Correlation is significant at P < 0.05*

SD: standard deviation; CV: coefficient of variation; GMI: glucose management indicator; TBR: time below range (< 70 mg/dl); TIR: time in range (70–180 mg/dL); TAR: time above range (> 180 mg/dL); MODD: mean of daily difference of blood glucose; LBGI: low blood glucose index; HBGI: high blood glucose index

Univariate and multivariate linear regression analyses were used to estimate the regression coefficients for adjusted SD HbA1c (Table 3). Furthermore, multivariate analysis with adjusted SD HbA1c as the dependent variable, and age, BMI, and the presence of hypoglycemia as the independent variables, identified the presence of hypoglycemia as the only significant determinant of adjusted SD HbA1c (β = 0.149, SE = 0.05, P = 0.007).

**Table 3:** Linear multivariable analysis with adjusted SD HbA1c as the dependent variable

	Univariable linear regression			Multivariable linear regression		
	β	SE	P	β	SE	P
Intercept				0.295	0.276	0.296
Sex, man/women	− 0.030	0.047	0.526			
Age, years	0.001	0.003	0.801	− 0.001	0.003	0.617
Height	0.002	0.003	0.511			
Weight	− 0.001	0.002	0.455			
BMI, kg/m^2^	− 0.007	0.006	0.232	− 0.005	0.006	0.422
SBP	0.001	0.001	0.513			
DBP	0.002	0.003	0.498			
Duration of diabetes	− 0.002	0.002	0.309			
Baseline HbA1c	− 0.014	0.042	0.731			
Creatinine	0.067	0.105	0.528			
eGFR	− 0.001	0.002	0.573			
Number of anti-diabetic agents	− 0.020	0.023	0.393			
Hypoglycemia, yes/no	0.135	0.042	0.004*	0.130	0.044	0.008*

We used univariable and multivariable linear regression analysis to estimate regression coefficients for adjusted SD HbA1c. The model fed into hypoglycemia. We selected one of the similar indicators from which multicollinearity may occur for each factor and examined in two models

Β: regression coefficient; SE: standard error; CI: confidence interval; BMI: body mass index; SBP: systolic blood pressure; DBP: diastolic blood pressure; eGFR: estimated glomerular filtration rate

### Factors associated with mean HbA1c

Table 2 shows the correlation between HbA1c and each factor. There were significant correlations between mean HbA1c and each of GMI (r = 0.624, P = 0.001), maximum (r = 0.467, P = 0.016), TAR (r = 0.525, P = 0.006), MODD (r = 0.570, P = 0.002), and HBGI (r = 0.452, P = 0.021; Fig. 1c). On the other hand, there was a negative correlation between mean HbA1c and TIR (r = − 0.445, P = 0.023; Fig. 1d).

### Hypoglycemia versus non-hypoglycemia

**Fig. 2:**
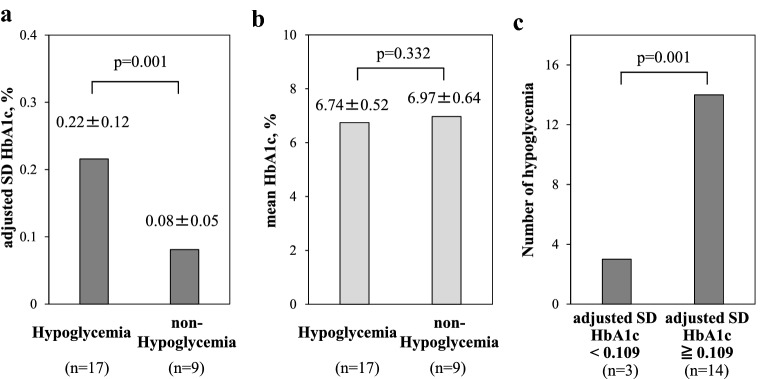
Comparison of patients with or without hypoglycemia. **a**, **b** adjusted SD HbA1c levels and mean HbA1c in patients with or without hypoglycemia. **c** Comparison of the number of hypoglycemic patients based on adjusted SD HbA1c cutoff value. The statistical difference was determined by the Wilcoxon signed rank test. SD: standard deviation

We examined the difference between adjusted SD HbA1c and mean HbA1c with and without hypoglycemia (Fig. 2a, b). Adjusted SD HbA1c was significantly higher in the hypoglycemic group (p = 0.001) at 0.22 ± 0.12%, compared to the non-hypoglycemic group at 0.08 ± 0.05%. On the other hand, there was no significant difference in mean HbA1c between with or without hypoglycemia.

In order to evaluate the cutoff of adjusted SD HbA1c in the hypoglycemic group, a ROC curve was constructed and the area under the curved surface was calculated. ROC curve analysis showed a cutoff value of SD HbA1c of 0.109% (area under the curve = 0.895, 95% CI 0.673–0.973) in the hypoglycemic group. Comparison of the number of patients with hypoglycemia below or above the adjusted SD HbA1c cutoff value showed significantly higher number of hypoglycemic patients in the adjusted SD HbA1c ≥ 0.109% group (p = 0.001, Fig. 2c).

## Discussion

We conducted a pilot study on HbA1c variability and CGM indexes, and showed that adjusted SD HbA1c is associated with the hypoglycemic indexes of TBR and LBGI. Although the association between HbA1c and CGM indexes has been analyzed previously [21, 22], this is the first study that dissected the association between HbA1c variability and CGM indexes.

Variations in blood glucose levels are known to be involved in the progression of diabetes vasculopathies, and several studies reported the association of fasting blood glucose variability and postprandial blood glucose with vascular complications.

HbA1c variability has also attracted attention in recent years, with SD and CV used as scales of HbA1c variability. Bouchi et al. [13] reported that SD HbA1c, which is independent of the common cardiovascular risk factors, is associated with the risk of onset of cardiovascular disease (CVD) in T2DM patients. Hirakawa et al. [14] reported that high SD HbA1c is associated with increased risk of onset of vascular events and increased mortality rate in patients of the ADVANCE trial intensive therapy group. Furthermore, Orsi et al. [15] reported that HbA1c variability in T2DM patients is a strong independent predictor of all-cause mortality in T2DM. Although these reports have shown that HbA1c variability is associated with mortality due to diabetes vasculopathies, the significance of HbA1c variability remains unknown. This study is the first to show that HbA1c variability is associated with CGM-based hypoglycemic indexes. Since hypoglycemia is known to be associated with diabetic vascular complications and related mortality risk, future studies are expected to report on the association of HbA1c variability with risk of cardiovascular mortality.

The objective of glycemic control is to normalize blood glucose level. The United Kingdom Prospective Diabetes Study (UKPDS) [23] obtained data that confirmed the so-called legacy effect associated with intensive glycemic control; therefore, if that state can be maintained, then a long-term prognosis can be expected. On the other hand, intensive glycemic control also increases the risk of hypoglycemia. Particularly severe hypoglycemia must be avoided, as it is associated with poor prognosis. HbA1c is a useful index for evaluating hyperglycemia; however, since HbA1c cannot be used to evaluate hypoglycemia, it is recommended to set a lower limit blood glucose for HbA1c according to the conditions and risk of hypoglycemia [24, 25]. Our study showed that high HbA1c variability increases the risk of hypoglycemia, and provided the cutoff value for adjusted SD HbA1c, above which the risk of hypoglycemia is increased. Our results call for evaluation of hypoglycemia in patients with high SD HbA1c.

As mentioned previously [4, 7], correction of HbA1c is required in order to prevent microangiopathies. In agreement with previous studies, our results showed that the mean HbA1c was associated with the CGM indexes of TIR [26], average glucose [8] and GMI [16]. Although the mean HbA1c was also associated with hyperglycemic indexes, such as maximum, TAR and HBGI, it was not associated with markers of hypoglycemia risk. It is not possible to determine the presence of hypoglycemia by HbA1c values alone; therefore, HbA1c target values should be set individually, taking into consideration the risk of hypoglycemia and support systems.

There were two limitations to this study. The first is we were unable to measure glucose density of ≥ 500 mg/dL in FGM; therefore, as per the decision of the primary physician, patients within the FGM range were recruited for this study. As such, our study did not include patients with poor glycemic control, and thus the results of such patients may be different to those obtained in this study. The second limitation is this was a cross-sectional study conducted at two facilities, and included a relatively small number of patients. Due to the small sample size in this study, it was not possible to adjust for confounding factors, such as renal function and diabetes medication. It was also difficult to include markers of hypoglycemia, such as TBR and LBGI, in the multivariate model. This study is a pilot study and the sample size is small. We plan to conduct a larger, multicenter study in the near future.

## Conclusions

We reported for the first time in this study that HbA1c variability is associated with the risk of hypoglycemia (as determined by CGM indexes). It has been reported that HbA1c variability is associated with vascular complications and mortality risk, and hypoglycemia is suggested as a possible contributory factor. For patients with high HbA1c variability, we recommend evaluation of hypoglycemia and adjustment of their treatment regimen, including their glucose-lowering agents.

## Supplementary Information

**Additional file 1: Table S1.** Differences in adjusted SD HbA1c by medication use.

## Data Availability

The datasets used and/or analysed during the current study are available from the corresponding author on reasonable request.

## Abbreviations

CGM: Continuous glucose monitoring
FGM: Flash glucose monitoring
TIR: The time in range
T2DM: Type 2 diabetes mellitus
AG: Average glucose level
SD: Standard deviation
CV: Coefficient of variation
TAR: Time above range
TBR: Time below range
GMI: Glucose management indicator
MODD: Mean daily difference of blood glucose
LBGI: Low blood glucose index
HBGI: High blood glucose index
eGFR: Estimated glomerular filtration rate
AUC: Area under the curve
BMI: Body mass index
DPP-4 inhibitor: Dipeptidyl peptidase-4 inhibitor
95% CI: 95% Confidence interval
CVD: Cardiovascular disease
