# Supplementary material for: Relationship between glycemic intraday variations evaluated in continuous glucose monitoring and HbA1c variability in type 2 diabetes: pilot study
Source: Diabetol Metab Syndr. 2021 Apr 15;13:45. doi: 10.1186/s13098-021-00663-2 (PMC8048042; doi:10.1186/s13098-021-00663-2)
Supplement: Supplementary file 1 — Additional file 1: Table S1. Differences in adjusted SD HbA1c by medication use. [file 13098_2021_663_MOESM1_ESM.doc]

**Table S1. Differences in adjusted SD HbA1c by medication use.**

|  | adjusted SD HbA1c (%) | |  |
| --- | --- | --- | --- |
|  | not used | used | p value |
| Glinide | 0.174±0.123 | 0.115±0.021 | 0.596 |
| DPP-4 inhibitor | 0.175±0.118 | 0.166±0.123 | 0.855^a^ |
| Biguanide | 0.186±0.150 | 0.150±0.071 | 0.429^a^ |
| Thiazolidine | 0.184±0.126 | 0.109±0.057 | 0.213^a^ |
| SGLT-2 inhibitor | 0.160±0.116 | 0.195±0.134 | 0.402 |
| α-glucose inhibitor | 0.178±0.131 | 0.133±0.037 | 0.191^a^ |
| GLP-1 receptor | 0.163±0.117 | 0.219±0.145 | 0.446 |
| Insulin | 0.169±0.124 | 0.172±0.032 | 0.500 |

Data are mean±SD. P values are for differences between the presence or absence of drug use.

^a^; The statistical significance of differences in mean values between two groups was assessed with Student’s t test if variances were equal as determined by an F test, and otherwise with Welch’s test.

Unmarked, Mann-Whitney test was used.

SD, standard deviation; DPP-4 inhibitor, dipeptidyl peptidase-4 inhibitor; SGLT-2 inhibitor, sodium-glucose transporter-2 inhibitor; GLP-1 receptor, Glucagon-like peptide-1 receptor.
